# Divergent cis*-*regulatory haplotypes at *Tlr2* are associated with immune responsiveness

**DOI:** 10.1093/molbev/msag113

**Published:** 2026-04-29

**Authors:** Mridula Nandakumar, Max Lundberg, Mehrnaz Nouri, Christine Valfridsson, Fredric Carlsson, Lars Råberg

**Affiliations:** Department of Biology, Lund University, Kontaktvägen 10, Lund 223 62, Sweden; Department of Biology, Lund University, Kontaktvägen 10, Lund 223 62, Sweden; Department of Biology, Lund University, Kontaktvägen 10, Lund 223 62, Sweden; Department of Biology, Lund University, Kontaktvägen 10, Lund 223 62, Sweden; Department of Biology, Lund University, Kontaktvägen 10, Lund 223 62, Sweden; Department of Biology, Lund University, Kontaktvägen 10, Lund 223 62, Sweden

**Keywords:** allelic imbalance, evolutionary immunology, *Myodes glareolus*, pathogen-mediated selection, Toll-like receptor

## Abstract

Positive and balancing selection on pattern recognition receptors (PRRs) is widely thought to target ligand-binding domains and affect the specificity of recognition of different pathogens. Alternatively, positive/balancing selection on PRRs could affect general responsiveness by targeting for example signaling domains or cis*-*regulatory variation. Studies of a wild rodent (the bank vole, *Clethrionomys glareolus*) have shown that *Tlr2*—a lipoprotein-binding PRR—is highly polymorphic with divergent haplotypes and signatures of balancing selection and that *Tlr2* genotype is associated with susceptibility to *Borrelia afzelii* infection in the wild. To investigate what aspect of TLR2 function has been under selection, we here perform integrated population genetic and functional analyses. Ex vivo infection experiments show that the protective *Tlr2* haplotype produces a stronger proinflammatory response to *B. afzelii* compared to the haplotype associated with susceptibility. *Tlr2* genotype has a similar, albeit not statistically significant, effect on responsiveness to the phylogenetically distant pathogen *Streptococcus pyogenes*. We find that the strongest signature of balancing selection is 4.6 kb upstream of the *Tlr2* CDS, near a putative enhancer, and that *Tlr2* exhibits allele-specific expression such that the protective haplotype is more expressed. Collectively, these results indicate that balancing selection has primarily acted on cis*-*regulatory variation affecting the general responsiveness via TLR2 signaling rather than on polymorphisms affecting TLR2 ligand-binding specificity.

## Introduction

Pattern recognition receptors (PRRs) play a crucial role in initiating immune responses against invading pathogens by recognizing molecular structures unique to microbes, so-called pathogen-associated molecular patterns (PAMPs). The existence of PRRs was hypothesized by Charles Janeway in his seminal 1989 paper (Janeway 1989), and this led to an explosion of research on innate immune recognition. PRRs are now known to represent a universal mechanism of innate sensing of infection, occurring in both animals and plants (Pradeu et al. 2024). In vertebrates, several different families of PRRs have been described, of which Toll-like receptors (TLRs) have attracted most attention (Carroll et al. 2024).

Each PRR senses different types of PAMPs, often complex molecules such as lipopolysaccharides or lipopeptides (Pradeu et al. 2024). A basic tenet of Janeway's concept of pattern recognition is that a limited set of innate receptors is sufficient to sense infection by virtually any pathogen because the receptors recognize molecules that serve essential functions in microbes and therefore are highly conserved. According to this view, PRRs should be conserved too, that is, they should be under purifying (negative) selection, particularly the ligand-binding domains. Nevertheless, studies reporting positive or balancing selection on PRRs are accumulating, including both comparative and population genetic analyses (Sackton et al. 2007; Barreiro et al. 2009; Wlasiuk and Nachman 2010; Jostins et al. 2012; Grossman et al. 2013; Velová et al. 2018; Liu et al. 2020; Lundberg et al. 2020; Fiddaman et al. 2022). Given the original expectation that PRRs should be conserved, findings of positive/balancing selection raise the question of what aspect of PRR function has been the target of such selection.

The predominant idea is that because PRRs are involved in direct physical interactions with microbes, there can be antagonistic reciprocal selection between pathogen and host leading to coevolution, where PRRs evolve to track escape mutations in PAMPs (Sackton et al. 2007; Wlasiuk and Nachman 2010; Dybdahl et al. 2014; Lundberg et al. 2020; Minias and Vinkler 2022; Vinkler et al. 2023; Snoeck et al. 2025). Such selection should primarily target ligand-binding domains to affect specificity, and selection to enhance recognition of a PAMP from one pathogen might impair recognition of PAMPs from other pathogens so that there is a trade-off between recognition of different pathogens (e.g. lipopeptides from different strains/species of pathogens). Several comparative studies have found high rates of positive selection on codons in PRR ligand-binding domains, consistent with selection for specificity (Sackton et al. 2007; Velová et al. 2018; Liu et al. 2020), although the functional effects of the substitutions rarely are known. On the other hand, population genetic analyses of human PRRs have revealed recent positive or balancing selection targeting polymorphisms that likely influence general responsiveness (i.e. the ability of a PRR to induce a response to all pathogens carrying a cognate PAMP) rather than specificity. Examples include single nucleotide polymorphisms (SNPs) affecting TLR1 translocation to the cell surface (Barreiro et al. 2009), TLR5 dimerization (Grossman et al. 2013), and NOD2 protein integrity (Jostins et al. 2012). Thus, we lack a coherent understanding of how positive/balancing selection acts on PRRs, that is, whether such selection generally affects ligand-binding specificity or general responsiveness.

In the bank vole (*Clethrionomys glareolus*, previously *Myodes*), an abundant and widely distributed rodent in Europe and western Siberia (Wilson et al. 2017), the lipoprotein-binding Toll-like receptor 2 (TLR2) is a highly polymorphic PRR (Tschirren et al. 2013). The *Tlr2* coding sequence (CDS) has divergent haplotypes with frequencies that vary geographically (Tschirren et al. 2012; Morger et al. 2015). In our study population in southern Sweden, two haplotype clusters, c1 and c2, are common (Tschirren et al. 2013), and population genetic analyses indicate that the polymorphism is consistent with long-term balancing selection (Lundberg et al. 2020). An epidemiological study of bank voles found that *Tlr2* genotype is associated with infection by *Borrelia afzelii* (Tschirren et al. 2013), a tick-transmitted pathogen that has small mammals as its main reservoir hosts (Hanincová et al. 2003; Råberg et al. 2017). Specifically, adult bank voles homozygous for *Tlr2* c1 have ∼50% infection prevalence compared to ∼20% in those homozygous for c2, with heterozygotes (c1/c2) showing an intermediate phenotype (Tschirren et al. 2013). Here, we employ this system to gain insights into how natural selection has acted on a classical PRR. To this end, we combine population genetic analyses and ex vivo infection experiments to identify targets of selection and investigate functional consequences of *Tlr2* polymorphism.

## Results

### Polymorphism and selection in bank vole *Tlr2*

**Figure 1:**
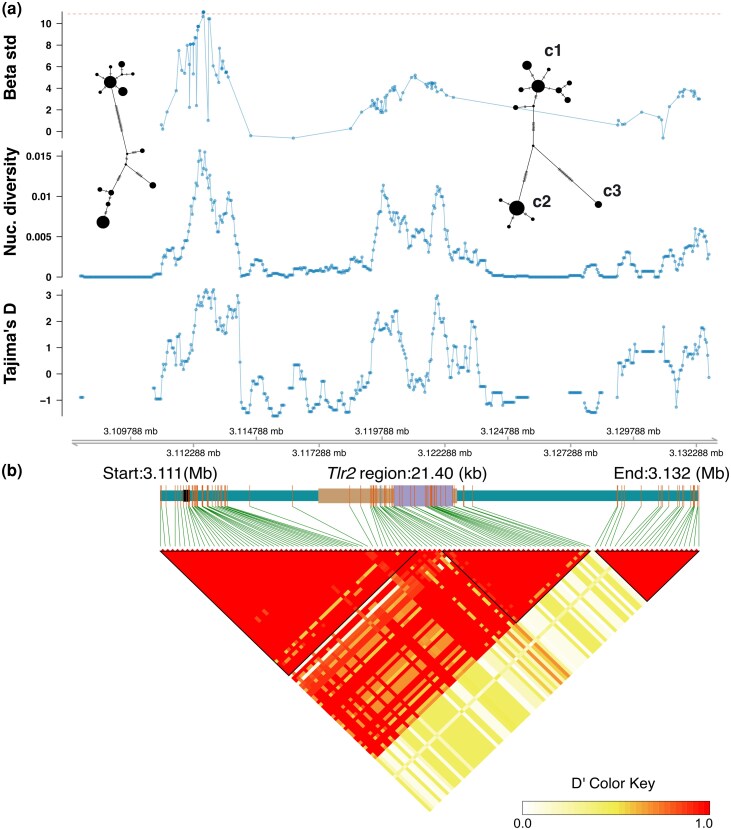
Population genetic analyses of bank vole *Tlr2* ± 10 kb, based on WGS data from 31 individuals. a) Beta for SNPs with MAF ≥ 0.15 (red dashed line indicates 95th percentile) and sliding window analyses of nucleotide diversity (π) and Tajima's D. Haplotype networks are for the polymorphic region ∼5 kb upstream of *Tlr2* (2,862 bp) and the CDS (2,352 bp). b) Haploview plot showing LD between all SNPs with MAF ≥ 0.15. Brown box is *Tlr2* gene, lilac box is CDS, and black box indicates blastn hit of mouse enhancer.

Sliding window analyses of population genomic data showed that several regions in and near *Tlr2* are highly polymorphic. Specifically, there are peaks in nucleotide diversity (π) and Tajima's D both in the single coding exon of *Tlr2* and in the intron between the exon and the 5′ UTR, as well as in a region ∼5 kb upstream of the gene (Fig. 1a). A previous analysis showed that the *Tlr2* CDS has a signature of balancing selection, as indicated by a HKA test (Lundberg et al. 2020). To test for signatures of balancing selection across both coding and non-coding sequences, we used Betascan2 (Siewert and Voight 2020). This showed that there were four SNPs 4.6 kb upstream of *Tlr2* with β scores in the 95th percentile, indicating that this region has been a target of balancing selection. This region also had the highest nucleotide diversity and Tajima's D values (Fig. 1a). Despite the significant HKA test for the CDS (Lundberg et al. 2020), the SNP in the CDS with the highest β score only reached the 70th percentile.

In our study population, the CDS has three divergent haplotype clusters (Fig. 1a), one of which (c3) is rare (6%) and not considered further here. The other two (c1 and c2) are separated by 17 SNPs, of which seven are missense. The frequencies of haplotype clusters c1 and c2 are 55% and 39%, respectively. TLR2 is expressed on the cell surface and consists of a N-terminal extracellular domain containing leucine-rich repeats that are involved in ligand binding and dimerization with the co-receptors TLR1/TLR6, a transmembrane region, and a C-terminal signaling domain (Toll-Interleukin-1 receptor homology; TIR). One missense SNP is in the signal peptide at the N terminus (which is responsible for directing TLR2 to the cell surface), and six are in the extracellular domain (Figure S1). Of the latter, one (in codon 275; Figure S1) is in the ligand-binding region, although not at the same position as residues directly involved in ligand interaction in mouse and human (Figure S1). The remaining five missense SNPs are in a region of the extracellular domain that is involved in neither ligand binding nor dimerization (Figure S1). A comparative analysis of *Tlr2* from 30 rodent species (Nandakumar et al. 2025) using HyPhy MEME (Murrell et al. 2012) showed there were eight codons with signatures of positive (i.e. diversifying) selection spread throughout the CDS (or 21 codons if the presence of multinucleotide substitutions is not taken into account) (Figure S1). None of the seven missense SNPs that separate *Tlr2* c1 and c2 in bank voles are in codons that are positively selected across rodent species (Figure S1).

The polymorphic region upstream of *Tlr2* has a similar haplotype network structure as the CDS (Fig. 1a). The corresponding region in the *Mus musculus* genome contains an enhancer (at position 3:83752157–83752802 in GRCm39). A blastn search against the bank vole genome using the *M. musculus* enhancer sequence as query strongly matched a region ∼5 kb upstream of bank vole *Tlr2* (Fig. 1b; Figure S2), suggesting that this is a regulatory region also in the bank vole.

The seven missense SNPs in the CDS that separate c1 and c2 are in linkage disequilibrium (LD) with the four SNPs with signatures of balancing selection 4.6 kb upstream of *Tlr2*, near the putative enhancer (*r*^2^: 0.35 to 0.56; D′: 0.69 to 1; Fig. 1b; Figure S2).

Thus, these analyses show that the strongest signature of balancing selection is near a putative cis*-*regulatory region and that SNPs in this region are in LD with SNPs in the CDS previously found to be associated with *B. afzelii* infection (Tschirren et al. 2013).

### Ex vivo infection experiments and immune responses

To investigate how *Tlr2* genotype affects immune responsiveness and whether effects are pathogen-specific, we performed ex vivo infection experiments with two different bacterial pathogens, *B. afzelii* and *Streptococcus pyogenes*. Both species contain lipoproteins and should therefore trigger immune responses by TLR2 signaling (Farhat et al. 2008). The bank vole is one of the most important reservoir hosts for *B. afzelii* (Råberg et al. 2017). *Streptococcus pyogenes* is a human-specific pathogen, but other uncharacterized β-hemolytic bacteria in the genus *Streptococcus* have been isolated from the upper respiratory tract of bank voles in our study population (K. Wollein Waldetoft pers. comm.). We used *B. afzelii* as one of the challenge pathogens in the infection experiment as *Tlr2* genotype is associated with *B. afzelii* infection (Tschirren et al. 2013) and *S. pyogenes* as a model pathogen that is phylogenetically distant from *B. afzelii*, yet signaling through TLR2. This allowed us to explore whether any effects of *Tlr2* genotype are specific to *B. afzelii* or applies to other pathogens too. In particular, we are interested in whether there are any indications that *Tlr2* genotype has opposing effects on responses to the two bacteria, that is, a genetic trade-off.

**Figure 2:**
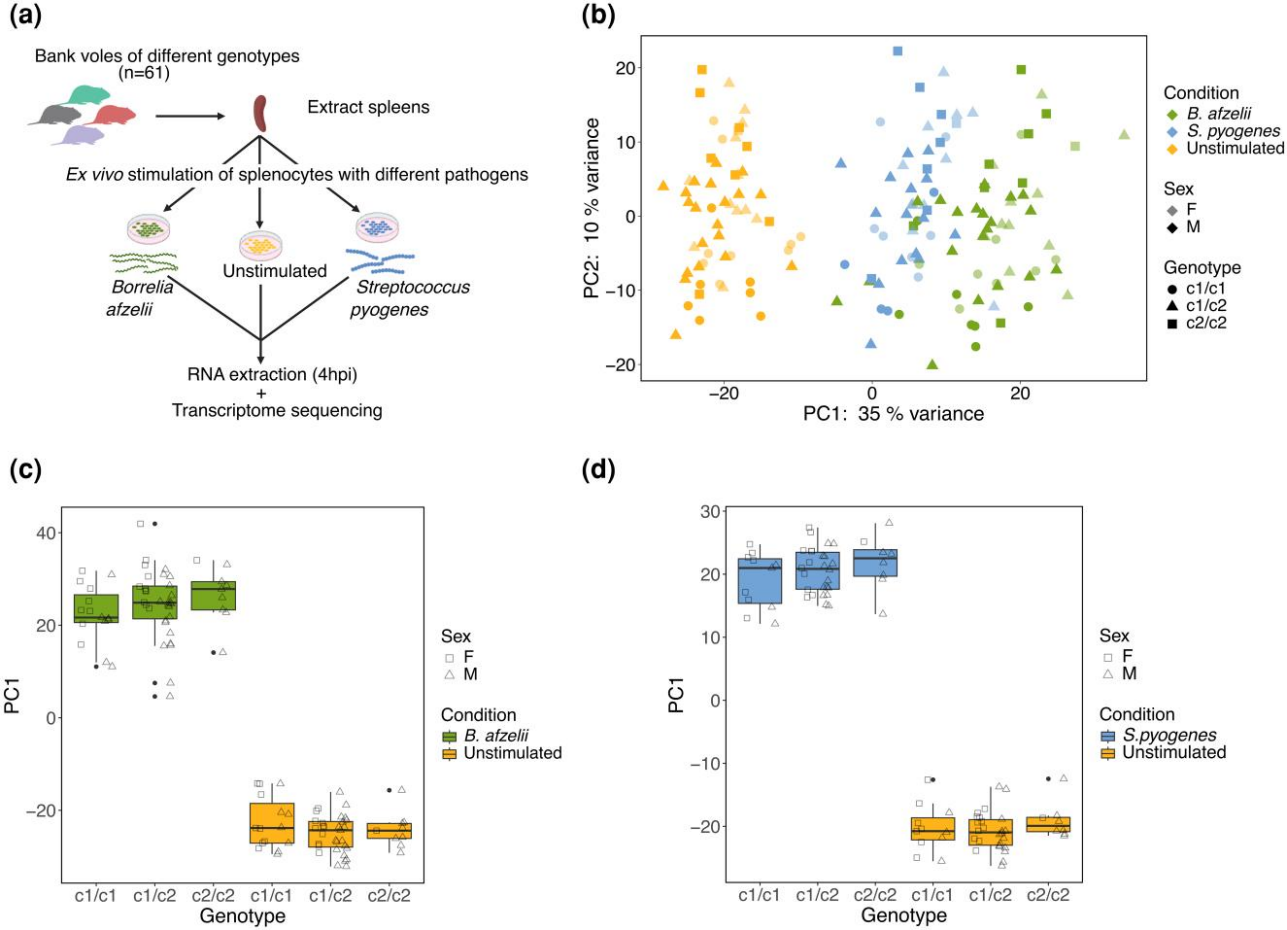
Ex vivo immune responses to *B. afzelii* and *S. pyogenes.* a) Experimental setup. Created in BioRender.com. b) PCA of all samples. c) Immune response of different *Tlr2* genotypes to *B. afzelii* as measured by all DEGs. d) Immune response of different *Tlr2* genotypes to *S. pyogenes* as measured by all DEGs. The symbols for F and M are on the left and right side of each boxplot, respectively.

Splenocytes from bank voles were stimulated with live *B. afzelii* and *S. pyogenes* or left unstimulated for 4 h whereafter RNA was extracted (Fig. 2a). We obtained transcriptome information from 57 individuals (Table S1). Sample sizes differ between treatment conditions because there was not always enough splenocytes available for three conditions (in those cases, *B. afzelii* infected and uninfected conditions were prioritized) and because extracted RNA was sometimes not of sufficient quality. Only samples with >25 M reads were retained, and the number of mapped reads averaged at 38 M per sample (86% of reads uniquely mapped on average).

A principal component analysis (PCA) showed that treatment conditions separated along PC1 but not PC2 (Fig. 2b). Treatment explained less variation than is typical in similar studies in humans (e.g. Quach et al. 2016); this might be an effect of that a proportion of the voles had (unknown) infections with other pathogens, which can affect both baseline expression and response to stimulation. *Borrelia afzelii* samples were more distant from controls on PC1 than were *S. pyogenes* samples (Fig. 2b). To investigate what kind of response was induced by *B. afzelii* and *S. pyogenes* infections, we performed differential gene expression analysis in DESeq2 using only treatment condition as factor. There were 1,501 and 1,102 differentially expressed genes (DEGs) in response to infection with *B. afzelii* and *S. pyogenes*, respectively (Figure S3a). Gene Ontology (GO) term enrichment analysis showed that most terms are shared between the two infections and are immune response-related (Figure S3b). Taken together, the PCA and analyses of DEGs indicated that the two pathogens induced qualitatively similar responses, but that the response to *B. afzelii* was somewhat stronger in the present setting.

### Effects of *Tlr2* genotype on immune responses

To investigate how *Tlr2* genotype affects immune responses, we performed general linear mixed models (GLMMs) separately for each stimulation, that is, *B. afzelii* vs unstimulated or *S. pyogenes* vs unstimulated. For each analysis, we only included cases where paired samples from individual bank voles were available (*N* = 55 for *B. afzelii* and *N* = 44 for *S. pyogenes*). In these analyses, we included the fixed factors condition (*B. afzelii*/*S. pyogenes* or unstimulated), *Tlr2* genotype (as a linear variable where the genotypes c1/c1, c1/c2, and c2/c2 were coded as −1, 0, and 1, respectively, i.e. testing for additive effects), sex (M or F), and their two- and three-way interactions. We also included vole individual as a random effect. The key term in the models is the *Tlr2* genotype × condition interaction, which tests if different genotypes respond differently to stimulation.

We first used PC1 scores from PCAs of DEGs in response to *B. afzelii* or *S. pyogenes* as measures of the immune responses. In the case of *B. afzelii*, there were significant effects of *Tlr2* genotype (*F*(1,104) = 8.91, *P* = 0.004) and *Tlr2* genotype × condition (*F*(1,52) = 6.53, *P* = 0.013; Fig. 2c). There were also significant effects of sex (*F*(1,104) = 18.1, *P* < 0.001) and sex × condition (*F*(1,52) = 5.58, *P* = 0.021) with females having stronger responses (Fig. 2c; see Table S2 for full model details). In case of *S. pyogenes*, there was an effect of *Tlr2* genotype in the same direction as for *B. afzelii* (Fig. 2d; F(1,42) = 4.65, *P* = 0.037), but *Tlr2* genotype × condition was not significant (*P* = 0.35; see Table S3 for full model details). As an alternative, we performed analyses of differential gene expression with *Tlr2* genotype as factor, separately for each condition. These analyses showed that DEGs between *Tlr2* genotypes in *B. afzelii*–stimulated splenocytes were enriched in immune response-related GO terms while this was not the case in unstimulated splenocytes, consistent with an effect of *Tlr2* genotype on the immune response to *B. afzelii* (Figure S4).

**Figure 3:**
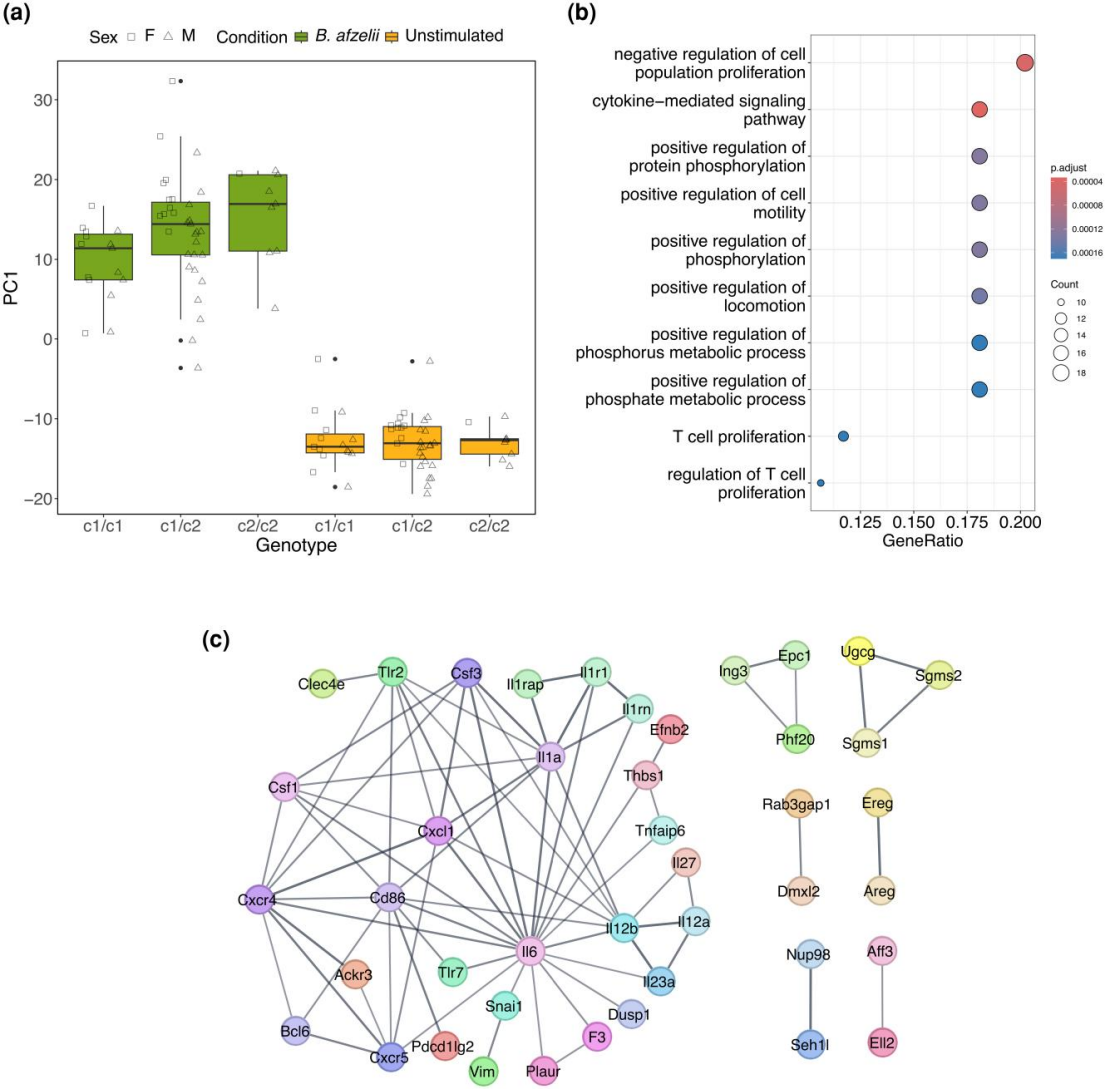
Analyses of the module of co-expressed genes (module 9) most strongly associated with *B. afzelii*-stimulation. a) Immune response of different *Tlr2* genotypes to *B. afzelii* as measured by module 9 genes. The symbols for F and M are on the left and right side of each boxplot, respectively. b) GO term enrichment of module 9 key genes. c) Protein–protein interaction network of module 9 key genes.

It seems likely that the effect of *Tlr2* genotype on the response to *B. afzelii* primarily affects a subset of DEGs, for example, those downstream of TLR2 signaling. To pinpoint such genes, we used network analysis (WGCNA) to identify modules of co-expressed genes (Langfelder and Horvath 2008). This yielded 23 modules containing 21 to 2,226 genes (Figure S5). The module most strongly associated with *B. afzelii* stimulation was module 9 (*n* = 384 genes; Figure S5). It contained *Tlr2* and other genes showing expression patterns correlated with *Tlr2*, that is, potentially downstream effects of TLR2 signaling. GO term enrichment analysis revealed that module 9 genes were associated with cell proliferation and differentiation, cytokine signaling, and chemotaxis (Figure S6). We repeated the analysis of effects of *Tlr2* genotype on the response to *B. afzelii* using PC1 scores of module 9 genes as a measure of the immune response. With this subset of genes, the effect of *Tlr2* genotype × condition was stronger as compared to the analysis based on all DEGs (*F*(1,52) = 8.65, *P* = 0.0049; Fig. 3a; see Table S4 for full model details).

To further narrow down the genes affected by *Tlr2* genotype in response to *B. afzelii*, we focused on highly significant module 9 genes that heavily influenced the expression profile for the module and strongly associated with *B. afzelii* infection (i.e. genes with >0.7 Pearson correlation with module 9 eigengene and *B. afzelii* infection), resulting in 104 key genes. These were enriched in GO terms related to cell proliferation, cytokine signaling, and protein phosphorylation (Fig. 3b). A protein–protein interaction network constructed using STRING (Szklarczyk et al. 2023) showed that central nodes include the proinflammatory cytokines Il-6, Il-12β, and Il-1α, the chemokine Cxcl1 (which recruits neutrophils), and the co-stimulatory signal Cd86 (Fig. 3c). A PCA with module 9 key genes showed that *Il6*, *Il12b*, *Il1a*, *Cxcl1*, and *Cd86* all had positive loadings on PC1 similar to *Tlr2*, and a GLMM with PC1 scores for module 9 key genes showed a significant effect of *Tlr2* genotype × condition (*F*(1,52) = 10.3, *P* = 0.002), with *Tlr2* c2 inducing a stronger response.

We next considered the possibility that *Tlr2* genotype has opposing effects on specific components of the responses to *B. afzelii* and *S. pyogenes*, as identified by WGCNA. For this, we considered the modules that were associated with stimulation (20 modules; Figure S5). Module 9, which was strongly associated with *B. afzelii* stimulation (see above), was also associated with *S. pyogenes* stimulation (Figure S5). The effect of *Tlr2* genotype on the response of module 9 genes to *S. pyogenes* was in the same direction as for *B. afzelii*, although the term *Tlr2* genotype × condition was not significant (*F*(1,43) = 2.81, *P* = 0.1; Figure S7). Another module of interest is module 2, which contained 1,471 genes enriched in GO terms related to leukocyte activation, etc. (Figure S6), and was associated with both *B. afzelii* and *S. pyogenes* infection (Figure S5). As with module 9, the direction of the effect of *Tlr2* genotype was similar for the responses to *B. afzelii* and *S. pyogenes*, although *Tlr2* genotype × condition was not significant for any of the responses (*P* = 0.069 and *P* = 0.43, respectively; Figure S7). The remaining modules that were associated with stimulation showed GO term enrichment less clearly related to immune response (Figure S6). In any case, for none of them was there any indication that the direction of the effect of *Tlr2* genotype on gene expression differed between *B. afzelii* and *S. pyogenes* infection (Figure S7). Overall, these analyses show that *Tlr2* genotype affects the immune response to *B. afzelii.* Effects of *Tlr2* genotype on the response to *S. pyogenes* were not significant, but there was no indication that *Tlr2* genotype had opposing effects on the responses to *B. afzelii* and *S. pyogenes*.

### Cis-regulatory variation

**Figure 4:**
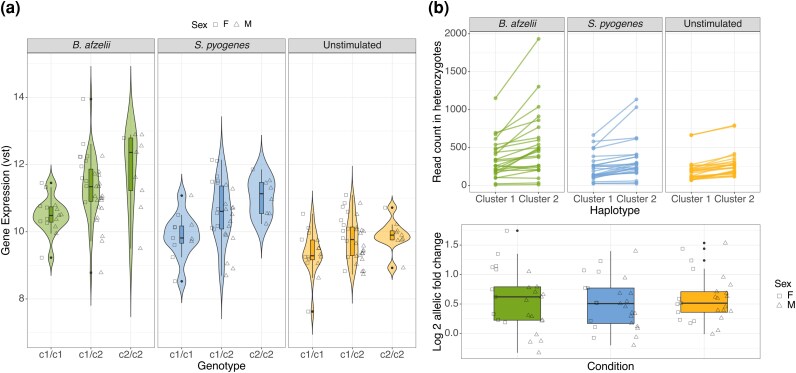
ASE of *Tlr2*. a) Expression of *Tlr2* in different *Tlr2* genotypes in different treatment conditions of the ex vivo infection experiment. The symbols for F and M are on the left and right side of each violin plot, respectively. b) Expression of *Tlr2* haplotype c1 and c2 in different conditions in *Tlr2* heterozygotes. Upper panel: read counts; lower panel: fold change.

The effect of *Tlr2* genotype on the response to *B. afzelii* could be an effect of polymorphisms in the CDS and/or cis-regulatory regions. The finding that the strongest signature of balancing selection was near a putative enhancer (Fig. 1a; Figure S2) suggests cis*-*regulatory variation might play an important role. Consistent with cis-regulatory variation, bank voles homozygous for c2 had higher expression of *Tlr2* than those homozygous for c1 in all conditions (GLMs with *Tlr2* genotype, sex, and their interaction: *B. afzelii*: *F*(1,52) = 16.2, *P* = 0.00001; *S. pyogenes*: *F*(1,41) = 9.50, *P* = 0.00014; uninfected: *F*(1,54) = 3.38, *P* = 0.0034; Fig. 4a). However, this pattern could also be an effect of polymorphism in the CDS in combination with positive feedback of TLR2 signaling on *Tlr2* expression.

To formally investigate if there is cis-regulatory variation, we tested for allele-specific expression (ASE, a.k.a. allelic imbalance) by comparing expression of c1 and c2 in individuals heterozygous for *Tlr2* (Fan et al. 2020; Cleary and Seoighe 2021). To this end, we estimated read counts separately for c1 and c2. *Tlr2* c2 was generally more expressed than c1 in each of the conditions, with log2 allelic fold change around 0.5 and significantly different from 0 in each condition (*t*-test, *P* < 0.0001; Fig. 4b), thus indicating ASE.

## Discussion

We found that bank vole *Tlr2* genotype affects the immune response to *B. afzelii*. Specifically, the haplotype associated with lower infection prevalence in wild bank voles induces a stronger transcriptional response involving for example the proinflammatory cytokine *Il6* and the neutrophil-recruiting chemokine *Cxcl1*. This represents a textbook response to extracellular bacteria, thus providing a likely explanation to the difference in susceptibility to *B. afzelii* infection between the genotypes seen in the wild (Tschirren et al. 2013). Importantly, even though the *Tlr2* CDS is highly polymorphic, the main target of balancing selection appears to be a cis-regulatory element. Consistent with this interpretation, there was allele-specific expression of *Tlr2*. Effects of *Tlr2* genotype on the response to *S. pyogenes* were generally in the same direction as for *B. afzelii*, although not statistically significant. We found no indication of a trade-off between the responses to *B. afzelii* and *S. pyogenes*, neither for the overall response nor for any of the modules of co-expressed genes, although there could of course be trade-offs involving other pathogens. Taken together, these results indicate that selection on bank vole *Tlr2* has primarily targeted regulatory variation and maintains polymorphism that affects general responsiveness rather than specificity of ligand binding.

The nature of selection on bank vole *Tlr2* thus appears to be similar to what has been found for some human PRRs—including *TLR1*, *TLR5*, and *NOD2*—insofar that it affects general responsiveness rather than specificity. The evolutionary modifications of general responsiveness of these PRRs have, however, been achieved through different mechanisms, with selection on SNPs affecting cell surface translocation (*TLR1*) (Barreiro et al. 2009), dimerization (*TLR5*) (Grossman et al. 2013), protein integrity (*NOD2*) (Jostins et al. 2012), and gene expression (*Tlr2*; present study). It is noteworthy that previous studies have found positive/balancing selection in the CDS of PRR genes, whereas in bank vole *Tlr2* balancing selection appears to have primarily targeted cis-regulatory variation. Balancing selection on cis*-*regulatory variation has, however, been reported for several other types of immune genes, including human *DEFB1* (Cagliani et al. 2008), *HLA-G* (Gineau et al. 2015), and *CCR5* (Bamshad et al. 2002).

Polymorphisms affecting general responsiveness could be maintained by a trade-off with some other trait, especially in combination with spatial or temporal heterogeneity in selection. In *Arabidopsis*, polymorphism for general resistance has been shown to be maintained through a trade-off with growth (Todesco et al. 2010). In case of vertebrate immunity, a potentially important trade-off is between resistance to pathogens and susceptibility to inflammatory/autoimmune disease (Quach et al. 2016). Indeed, there are several examples of such trade-offs in humans, including for the PRR *IFIH1* (Gorman et al. 2017), although virtually nothing is known about whether costs in the form of inflammatory/autoimmune disease play a role in short-lived animals like rodents. Maintenance of polymorphism by a trade-off on its own is facilitated if there is “reversal of dominance,” as this leads to heterozygote advantage (Grieshop et al. 2021). However, both the present ex vivo experiment and the epidemiological study (Tschirren et al. 2013) indicate that effects of *Tlr2* genotype on susceptibility and immune responsiveness are largely additive, suggesting that spatial or temporal heterogeneity in selection is also involved, for example, because of variation in overall pathogen abundance (Fumagalli et al. 2011; Levy et al. 2020).

Given that the cis*-*regulatory region upstream of *Tlr2* appears to be the main target of selection, the polymorphisms in the *Tlr2* CDS could be a result of hitchhiking as balancing selection increases neutral variation in surrounding regions (Charlesworth 2006). Alternatively, SNPs in both the cis*-*regulatory region and the CDS could have phenotypic effects and have been under selection. The LD between the cis*-*regulatory region and the CDS could then be a result of epistatic selection (Loisel et al. 2006). None of the missense SNPs in the CDS of bank vole *Tlr2* are at positions involved in ligand binding or other functions, as determined in *M. musculus* and human (Jin et al. 2007). Nevertheless, a study of human *TLR2* showed that an SNP in the extracellular domain but outside positions involved in ligand binding or dimerization had an effect on downstream signaling (Ben-Ali et al. 2011). Thus, also SNPs outside known functional sites can clearly have phenotypic effects, but whether any of the SNPs in the CDS of bank vole *Tlr2* affect function remains to be investigated.

PRR-mediated recognition of PAMPs is only one of several mechanisms of innate sensing of infection in animals and plants; others include missing-self recognition and effector-triggered immunity (Pradeu et al. 2024). Outside PRRs sensu stricto, there are examples of positive/balancing selection affecting both specificity and general responsiveness of innate sensors. For example, *RCR3* in *Solanum peruvianum* (a wild tomato), which encodes a “guardee” involved in effector-triggered immunity by recognizing a fungal virulence factor, has been under balancing selection affecting general responsiveness (Hörger et al. 2012). In contrast, *CEACAM3*, which encodes a phagocytic receptor in primates that binds bacterial adhesins, has been under strong positive selection affecting specificity for different bacteria (Adrian et al. 2019). The relative importance of selection on specificity vs general responsiveness for different types of innate sensors is an interesting topic for future research.

We found strong and consistent effects of sex on immune responses, with females having stronger responses. Sex differences in immune responses are common, but given they are to a large extent caused by sex hormones (Sharma et al. 2025), we were surprised to see such strong effects in a sexually monomorphic species, outside the reproductive season, and in an ex vivo setting. Nevertheless, these results fit with the previous epidemiological study which found that males have slightly higher rates of *B. afzelii* infection (Tschirren et al. 2013).

In summary, we found that *Tlr2* genotype affects the immune response to *B. afzelii* in a manner that may explain the differential susceptibility to infection between genotypes (Tschirren et al. 2013). There was no indication for a *Tlr2-*mediated trade-off between responses to *B. afzelii* and another bacterium (*S. pyogenes*). Taken together with the findings that the main target of selection is near a putative cis*-*regulatory element and that *Tlr2* exhibits allele-specific expression, our results indicate that bank vole *Tlr2* is an example of adaptive polymorphism for general responsiveness. Unlike selection for PRR specificity, selection on general responsiveness is not in conflict with Janeway's original concept of pattern recognition.

## Methods

### Population genomics and selection analyses

Analyses were based on whole-genome resequencing (WGS) data from 31 bank voles from one population in southern Sweden (Lundberg et al. 2020). We used the same set of variants as in Nandakumar et al. (2025), which had been obtained from aligning the WGS data to the reference genome (GCF_902806735.1). To facilitate downstream analyses, we additionally filtered SNPs to be located only within callable intervals. To compute callable intervals, we used samtools 1.19 (Danecek et al. 2021) to calculate sample-specific coverage for each position and bedtools 2.31 (Quinlan and Hall 2010) to merge adjacent positions with a coverage within 1/3 and 1.5 of the sample-specific median into callable intervals. Next, we intersected all of the sample-specific callable intervals to obtain intervals where all of the samples were callable.

Analyses of long-term balancing selection were performed with BetaScan2 (Siewert and Voight 2020). To obtain outgroup information for the balancing selection analyses, we collected a male grey red-backed vole (*Craseomys rufocanus*) in Ammarnäs, northern Sweden (65.96°N, 16.21°E). High molecular weight DNA was extracted from spleen using a nanobind protocol (Circulomics). A library was prepared using a SMRTBELL Prep Kit 3.0 (Pacific Biosciences, CA, USA), which was sequenced on a single Revio SMRT cell (Pacific Biosciences) and resulted in 5,028,113 high-fidelity (HiFi) reads with a mean length of 14,167 bp. The HiFi reads were mapped to the bank vole reference genome using minimap v2.22 (Li 2018) and alignments were converted into sorted bam files using samtools. From the alignments, we calculated callable intervals based on the median coverage using the same approach as for the bank voles. The callable intervals from the grey red-backed vole were then intersected with the bank vole callable intervals to provide a universal set of callable intervals across the species. Within these intervals, we used BCFtools 1.19 (Danecek et al. 2021) to obtain genotypes for the grey red-backed vole at SNP positions in the bank vole. This data set was filtered to contain only positions with homozygous genotypes, which were used to polarize the alleles in the bank vole and led to a final set of 18.9 M bi-allelic SNPs. In addition to the polarized variants, we also used BCFtools to identify substitutions between the species. Betascan2 was run with the polarized SNP and substitution data as input and using a window size of 2,000bp, SNPs with a minor allele frequency of at least 0.15 and reporting a standardized β2 statistic. We also included a genome-wide estimate of theta (0.0017) calculated from the WGS data and a divergence time between the bank vole and the grey red-backed vole. The divergence time was estimated as an interspecies coalescence time (23.2) in ballet 1.0 (DeGiorgio et al. 2014) using the same data set and a theta of 0.0017. Percentiles for the beta values were calculated separately for SNPs on autosomal and X-linked scaffolds. Collapsed paralogues and copy number variation may generate high β values, but by using strict coverage filtering and removing variants for which heterozygotes have excessively low or high proportion of alternate allele reads (allele balance), their impact should have been minimized in this data set (Nandakumar et al. 2025). Note also that there is no indication of *Tlr2* copy number variation from Sanger or Illumina sequencing data (no sites with >2 alleles in an individual, 50:50 peak ratios at all heterozygous sites in electropherograms, no deviation from Hardy-Weinberg equilibrium and no deviation in coverage).

To analyze patterns of LD around *Tlr2*, we extracted all SNPs on the same scaffold and used beagle 5.5 (Browning et al. 2021) to infer haplotypes. This data was used as input to haploview (Barrett et al. 2005). We also used VCFtools to calculate pairwise LD between focal SNPs in *Tlr2* and the upstream region. Sliding window analysis for nucleotide diversity and Tajima's D was performed using the R package PopGenome, with window and step size of 500 and 50, respectively (Pfeifer et al. 2014). Haplotype networks were constructed with POPART (Leigh and Bryant 2015). Blastn searches with sequences of *M. musculus* cis*-*regulatory elements as queries were performed at https://www.ncbi.nlm.nih.gov with the bank vole assembly (GCF_902806735.1) as the target database. Positively selected sites in rodent *Tlr2* were inferred using the program MEME (Murrell et al. 2012), part of the HyPhy package (Kosakovsky Pond et al. 2020), based on orthologues from 30 species (Nandakumar et al. 2025).

### Ex vivo infection experiment and transcriptome sequencing

Bank voles were trapped at Revingehed, 20 km east of Lund, southern Sweden using live traps (Ugglan Special, Grahnab, Gnosjö, Sweden). Animals were trapped during November to February. To minimize previous exposure to *B. afzelii*, we focused on subadult voles, that is, individuals born in the autumn after the peak of the tick-season. Ear biopsies for DNA extraction were collected in 70% ethanol. Animals were euthanized and spleens were harvested in 1× PBS and stored on ice until further processing.

*Borrelia afzelii* cultures were prepared by growing the inoculum in BSK-H medium at 30 °C for at least 24 h and later incubated at 37 °C, 12 h prior to the start of the stimulation. Cells were pelleted by centrifuging at 10,000 rpm for 5 min. Bacterial cells were resuspended in 1× PBS and counted using dark field microscopy.

*Streptococcus pyogenes* cultures were grown overnight at 37 °C in 10 mL Todd-Hewitt broth supplemented with 0.2% yeast extract. Cells grown to the exponential phase were washed twice with D-PBS and resuspended in RPMI (supplemented with 5% heat inactivated bovine serum albumin).

Primary splenocyte cultures were prepared from isolated spleens as follows. Spleens were crushed and passed through a 40 μm cell filter to obtain a single cell suspension. Red blood cells were then depleted by treatment with a lysis buffer, and the reaction was arrested by addition of 5 mL 1× PBS. Cells were pelleted by centrifugation at 2,000 rpm for 5 min. The cells were resuspended in RPMI (supplemented with 5% heat inactivated bovine serum albumin), cell counts obtained using a Burker chamber. 2 × 10^6^ cells were then plated per well in a 96 well plate totaling at least 6 × 10^6^ cells per condition. The cells were then treated with either live *B. afzelii* at MOI 4, live *S. pyogenes* at MOI 1 or RPMI (unstimulated).

Four hours post stimulation, the cell cultures were harvested for RNA extraction. Splenocytes were spun down at 2,000 rpm for 10 min, at 4 °C and the supernatant discarded. Cells were resuspended with an appropriate volume of RLT buffer with 20 mm DTT and lysed by passing through a QiaShredder column (Qiagen). The flowthroughs were then treated as per the Qiagen RNEasy Plus extraction kit (Qiagen) and finally RNA dissolved in 30 mL nuclease-free water.

RNA quality was checked using Agilent Bioanalyzer (Agilent). RNA samples with RNA integrity number (RIN) > 8 and sufficient concentration were sent to SciLife Lab, Stockholm, Sweden. Library preparation for sequencing involved polyA selection of total RNA. Paired-end sequences with 2 × 150 bp cycles were generated on Illumina NovaSeq S4-300 v1.5, using two lanes.

Individual voles were genotyped at *Tlr2* using Sanger sequencing as described in (Tschirren et al. 2013). Briefly, DNA was extracted from ear biopsies following (Laird et al. 1991). Primers targeting a 1,173 bp region of *Tlr2* CDS was used for sequencing on a Genetic Analyzer 3500 (Applied Biosystems) following BigDye terminator (Applied Biosystems). Genotypes were called manually in Geneious Prime 2020.2.4 (Biomatters).

Individuals with active *B. afzelii* infections at the time of trapping were determined by real-time qPCR targeting the *flaB* gene in the bacteria (Råberg 2012), using DNA extracted from ear biopsies.

We obtained transcriptome information from 61 individuals. Of these, three individuals later turned out to be positive for natural infections of *B. afzelii* as determined by qPCR, and one individual possessed the rare c3 haplotype of *Tlr2*; these four individuals were removed from all analyses.

### Differential gene expression

Sequence quality was checked using FastQC (https://www.bioinformatics.babraham.ac.uk/projects/fastqc/). Low-quality regions and adaptor sequences were trimmed using Trimmomatic v0.39 (Bolger et al. 2014). Reads were mapped against the reference bank vole genome (NCBI: GCA_902806735) using STAR v2.7.11a in the two-pass mode (Dobin et al. 2013), with transcriptome alignments enabled (−quantMode TranscriptomeSAM). Reads were quantified using RSEM v1.3.1 (Li and Dewey 2011). As samples were processed over the course of a few months, read counts were corrected for batch effects, using date of experiment as factor, while accounting for biological variability due to stimulation condition, *Tlr2* genotype and sex using CombatSeq, part of the package sva (Leek et al. 2012; Zhang et al. 2020). Differential expression analysis was performed in R v4.2.1 (R Core Team 2022) using the package DESeq2 (Love et al. 2014). GO analysis was performed using the package clusterProfiler (Yu et al. 2012).

### Network analysis

We used weighted-correlation network analysis (WGCNA) in R (Langfelder and Horvath 2008) to identify modules of correlated genes. WGCNA clusters genes showing similar patterns of expression into a module and the expression pattern of genes in each module is represented by an eigengene (first principal component of the module). The association between the eigengene for each gene module and different traits (e.g. experimental condition) can then be checked with the help of correlation analysis to identify an unbiased network of co-expressed genes. Variance-stabilized read counts were supplied as input to WGCNA. The co-expression power value β was set to 10, after evaluating the fit of the scale-free topology network. Depth of clustering was left at the default value (i.e. deepSplit = 2). A signed network type and Pearson correlation was used to establish association between traits (coded as binary variables, contrasted against all levels for a trait) and the eigengene for each module.

### Allele-specific expression

Transcriptome mapping against a reference is often biased against reads containing the non-reference allele, as aligners may discard reads with the alternate allele due to poor mapping (Castel et al. 2015; Cleary and Seoighe 2021). To account for this, bias correction of STAR mapped transcriptome reads was performed using WASP (van de Geijn et al. 2015). In short, reads overlapping known variants as inferred from the WGS data were remapped using STAR, with the allele at each variant locus flipped to contain the other allele. Reads that failed to remap to the same location in the genome after flipping the allele were discarded and PCR duplicates were removed.

Most ASE tools also require genotype of the samples being analyzed. To get this information, we genotyped individual bank voles using the set of unique, bias-corrected RNA reads obtained in the previous step. Reads from different conditions of the same individual were pooled to increase the read depth. Variant calling was performed in parallel with GNU Parallel (Tange 2022) across all RNA samples with FreeBayes v1.3.2 (Garrison and Marth 2012), with coverage per site limited to 300 reads. Only variants that qualified the following filters were retained: (i) strand bias (SAF and SAR > 1), (ii) read placement around variant (RPR and RPL > 1), (iii) phred-score base quality (QUAL > 30), (iv) number of reads for genotype (minDP > 5), and (v) missing data at each locus (−max-missing 0.75). Variants were filtered and only biallelic SNPs retained using a combination of VCFtools v0.1.16 (Danecek et al. 2011) and BCFtools v1.19 (Danecek et al. 2021). As variant calling from RNA data can be riddled with false positives due to technical errors, we finally retained only those variant loci that matched variant calls from the WGS data (Nandakumar et al. 2025). With this genotype information along with the bias-corrected RNA reads, the number of reads accounting for each allele was quantified using ASEReadCounter (Castel et al. 2015), part of GATK v4.3.0, where the minimum base quality and minimum mapping quality was set to 10 and 255 (uniquely mapped reads), respectively.

To facilitate comparison between treatment conditions, we limited the analysis to individuals heterozygous at *Tlr2* (c1/c2) that had transcriptome information from all three conditions (*B. afzelii*-stimulated, *S. pyogenes*-stimulated, unstimulated controls; *n* = 25). We limited false-positive discovery of ASE due to lowly expressed regions by requiring that each SNP should be covered by ≥7 total reads and each allele has count ≥ 3. We chose 13 polymorphic sites (the 7 missense + 6 synonymous SNPs) that distinguish the two haplotype clusters at *Tlr2* and passed these filters to check for differences in cis-regulation on *Tlr2* expression between the haplotypes. We tested for ASE where the direction of the allelic imbalance was consistent across individuals, i.e. where the measured exonic SNP is assumed to be in LD with one or more unknown regulatory variants. Read counts were pooled across all SNPs for each haplotype within individuals and allelic log fold change for each haplotype and individual [AFC = log2(alternate allele (i.e. c2) read counts/reference (c1) allele read counts)] and mean AFC for each SNP across heterozygous individuals within a condition were calculated (AFC = 0 means no allelic imbalance). Significant deviation of the mean AFC from zero in each condition was tested using a single sample *t*-test.

### Statistical analyses

Statistical analyses were performed in R 4.4.2 (R Core Team 2022). GLMMs were performed using the lmer function, part of the package lme4 (Bates et al. 2015). GLMs were performed using the lm function. The significance of the linear models was checked using a χ² or *F*-test with Kenward-Roger adjusted residual df, using type-3 sum of squares with the ANOVA function, part of the car package (Fox and Weisberg 2019).

## Supplementary Material

msag113_Supplementary_Data

## Data Availability

Raw RNA reads are deposited at NCBI SRA (PRJNA1222872). Raw reads from grey red-backed vole genome sequencing are deposited at EBI ENA (PRJEB102860). Codes used for analysis are available at GitHub (https://github.com/lraberg/Tlr2exVivo and https://github.com/lraberg/BalancingSelectionBankVole).
