# Supplementary material for: Divergent cis-regulatory haplotypes at Tlr2 are associated with immune responsiveness
Source: Mol Biol Evol. 2026 Apr 29;43(5):msag113. doi: 10.1093/molbev/msag113 (PMC13202456; doi:10.1093/molbev/msag113)
Supplement: msag113_Supplementary_Data [file msag113_supplementary_data.pdf]

Supplementary Information

Divergent *cis*-regulatory haplotypes at *Tlr2* are associated with immune responsiveness

Mridula Nandakumar, Max Lundberg, Mehrnaz Nouri, Christine Valfridsson,
Fredric Carlsson, Lars Råberg

Table S1. Sample sizes for different conditions and *Tlr2* genotypes.

Condition	Sample size	*Tlr2* genotype	Sample size	Sex (F/M)
B. afzelii	55	c1/c1	15	8/7
		c1/c2	31	11/20
		c2/c2	9	1/8
S. pyogenes	45	c1/c1	11	7/4
		c1/c2	26	11/15
		c2/c2	8	1/7
Unstimulated	57	c1/c1	16	9/7
		c1/c2	32	12/20
		c2/c2	9	1/8

Table S2. GLMM of overall transcriptional response to *B. afzelii* (i.e. PC1 for all DEGs) against condition (*B. afzelii* vs unstimulated), *Tlr2* genotype (c1/c1, c1/c2 or c2/c2 coded as −1, 0 and 1), and their 2- and 3-way interactions. 3-way interaction deleted at P=0.94. Vole individual was included as a random effect (P=1).

Source	F	df	P
Condition	839.9	1, 52	<0.00001
Genotype	8.91	1, 104	0.0035
Sex	18.1	1, 104	<0.00001
Condition × Genotype	6.53	1, 52	0.014
Condition × Sex	5.56	1, 52	0.022

Table S3. GLMM of overall transcriptional response to *S. pyogenes* (i.e. PC1 for all DEGs) against condition (*S. pyogenes* vs unstimulated), *Tlr2* genotype (c1/c1, c1/c2 or c2/c2 coded as −1, 0 and 1), and their 2- and 3-way interactions. Interactions deleted at P≥0.34. Vole individual was included as a random effect (P= 0.12).

Source	F	df	P
Condition	3996	1, 44	<0.00001
Genotype	4.65	1, 42	0.037
Sex	4.57	1, 42	0.038

Table S4. GLMM of transcriptional response of module 9 genes to *B. afzelii* (i.e. PC1 for module 9 genes) against condition (*B. afzelii* vs unstimulated), *Tlr2* genotype (c1/c1, c1/c2 or c2/c2 coded as −1, 0 and 1), and their 2- and 3-way interactions. 3-way interaction deleted at P=0.25. Vole individual was included as a random effect (P=1).

Source	F	df	P
Condition	349.1	1, 52	<0.00001
Genotype	20.0	1, 104	<0.00001
Sex	21.2	1, 104	<0.00001
Condition × Genotype	8.65	1, 52	0.0049
Condition × Sex	4.33	1, 52	0.042

Supplementary Information

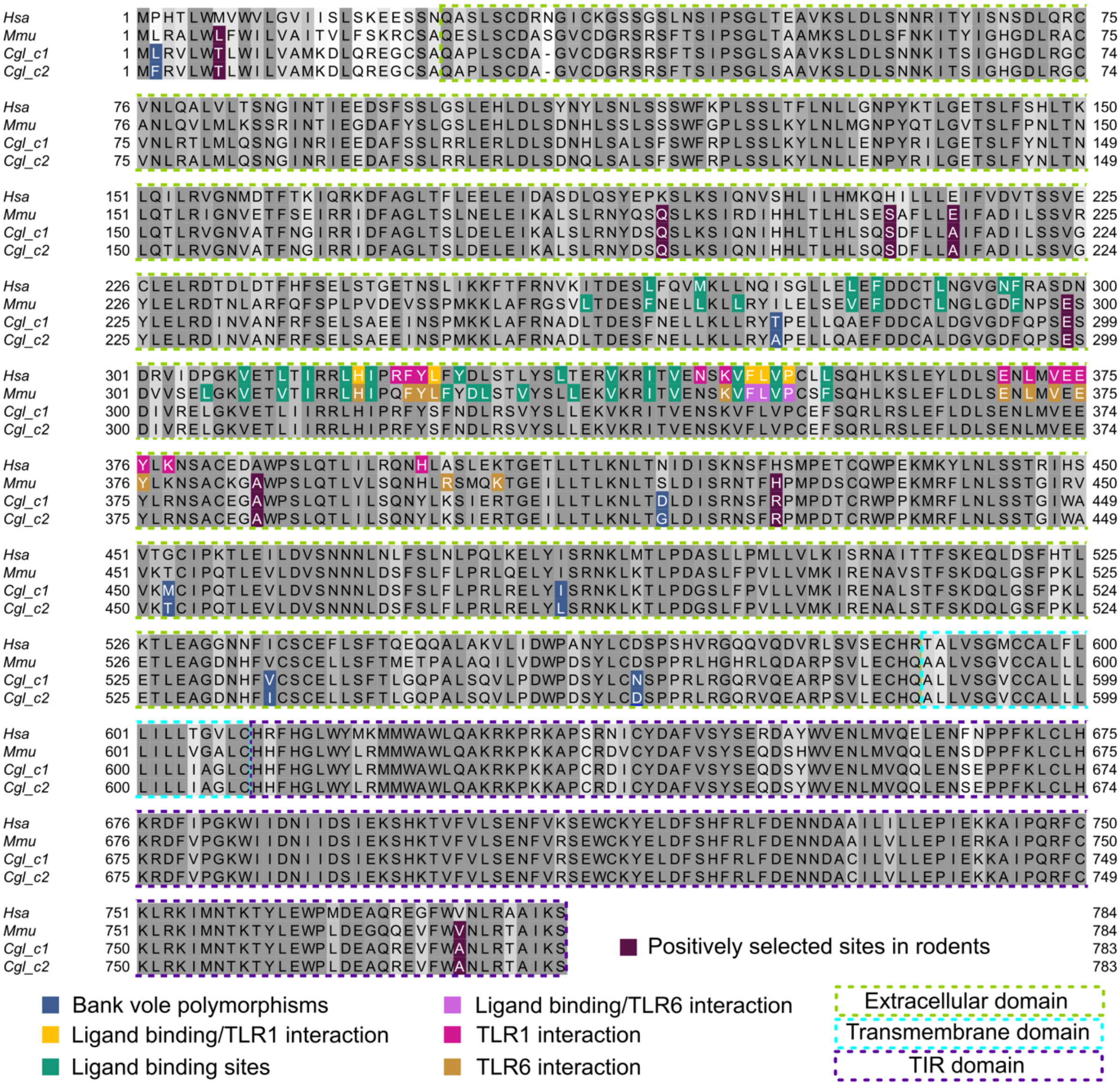

Figure S1. Alignment of human *TLR2* (Hsa), house mouse *Tlr2* (Mmu) and bank vole *Tlr2* (Cgl) c1 and c2. Positively selected sites in rodents were inferred from 30 species using the program MEME.

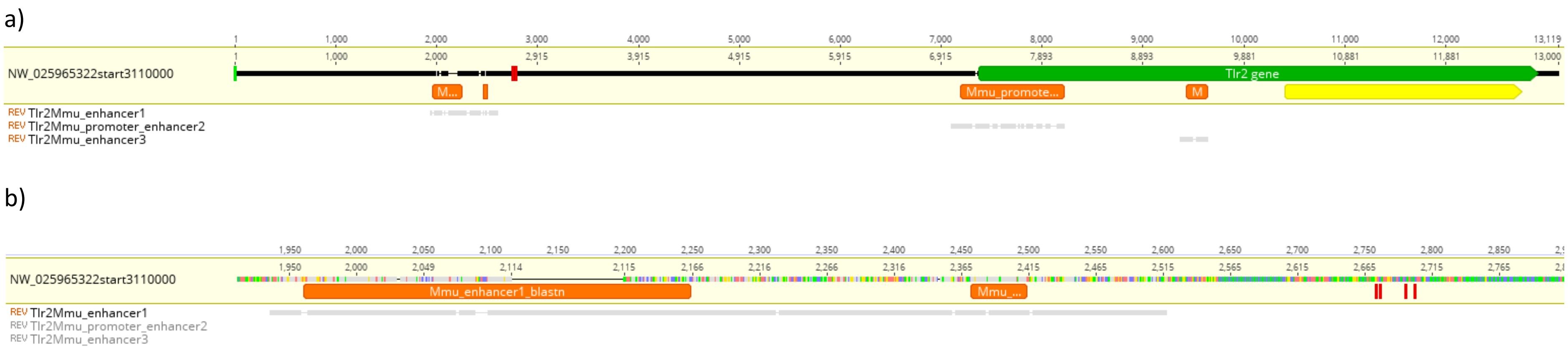

Figure S2. Regulatory elements of *Tlr2.* a) Bank vole *Tlr2* (green; CDS in yellow). Orange bars indicate blastn hits of enhancer and promoter sequences from *M. musculus.* Aligned full *M. musculus* enhancer and promoter sequences indicated below bank vole sequence. Red mark indicates the 4 SNPs with Beta values in 95th percentile. b) Zoom in of region with the 4 SNPs and the nearest enhancer.

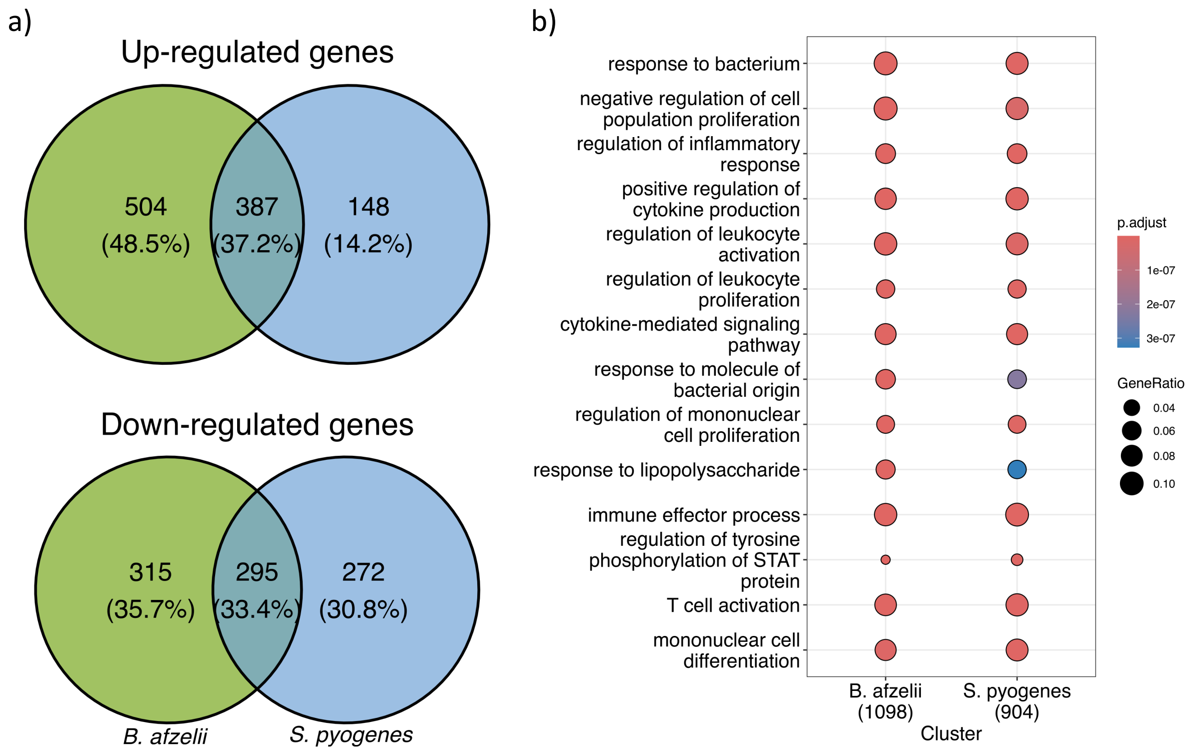

Figure S3. a) Number of differentially expressed genes (DEGs) at $\log_2$ FC $\geq|1|$ and $p_{adj}<0.05$ in response to *B. afzelii* and *S. pyogenes* that are up- or downregulated. b) Enrichment map of top 14 most significant gene ontology terms for each infection stimulation show mainly immune-related terms.

Supplementary Information

a)

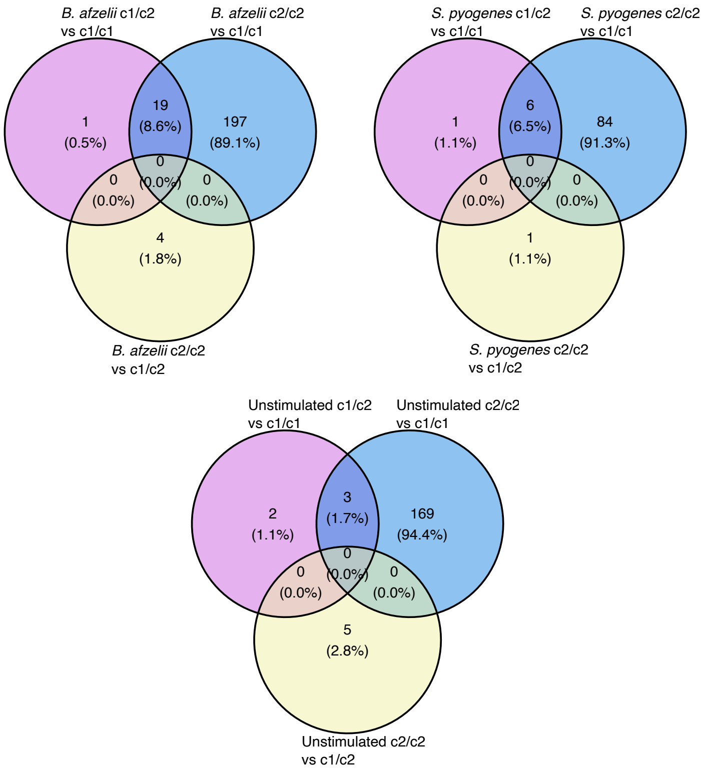

b)

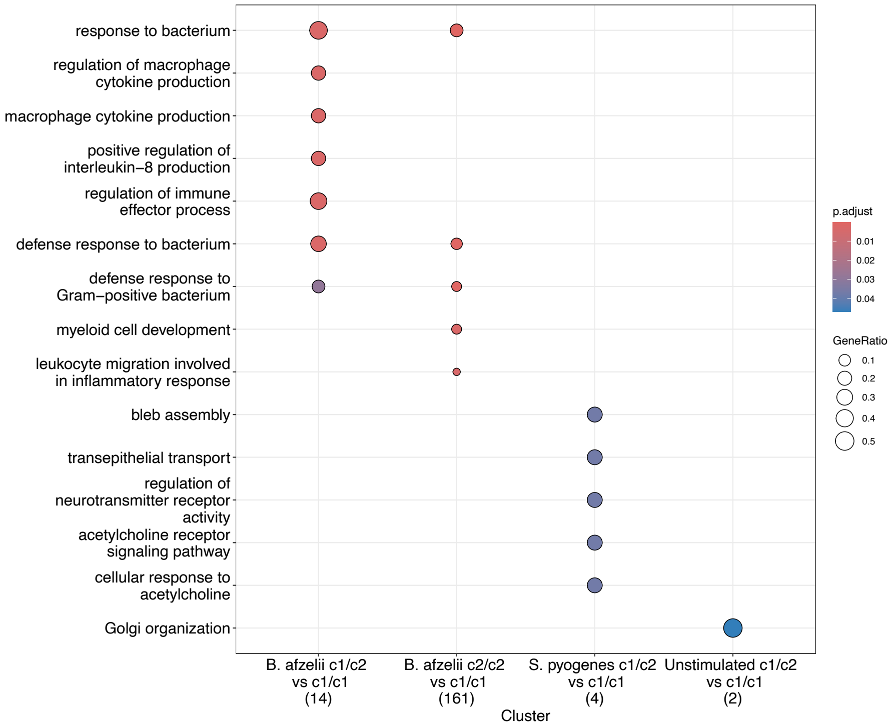

Figure S4. a) Overlap of differentially expressed genes (DEGs) at $\log_2$ FC $\geq|1|$ and $p_{adj}<0.05$ between *Tlr2* genotypes in different conditions. b) Enrichment map of significant gene ontology terms for DEGs between *Tlr2* genotypes in *B. afzelii*- and *S. pyogenes*- and un-stimulated splenocytes. Other comparison levels than those shown were not enriched for any term.

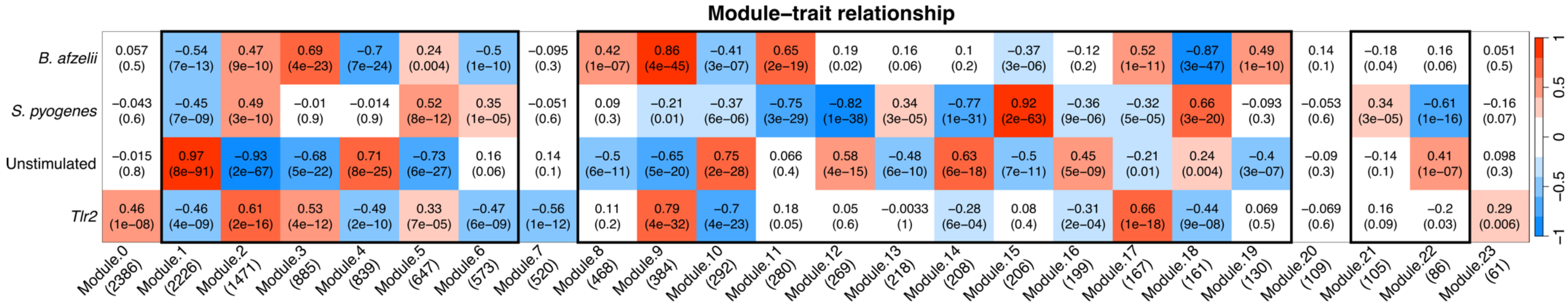

Figure S5. Heatmap of association between modules of coexpressed genes (identified with WGCNA) and condition, with colours indicating strength and direction of correlation. Correlation coefficient and its significance (in parenthesis) are indicated within each box. Number of genes belonging to each module is indicated below each module name. The modules that were associated with stimulation are indicated with black frames. GO term enrichment and effects of *Tlr2* genotype on these modules are shown in Fig S6 and S7, respectively.

Supplementary Information

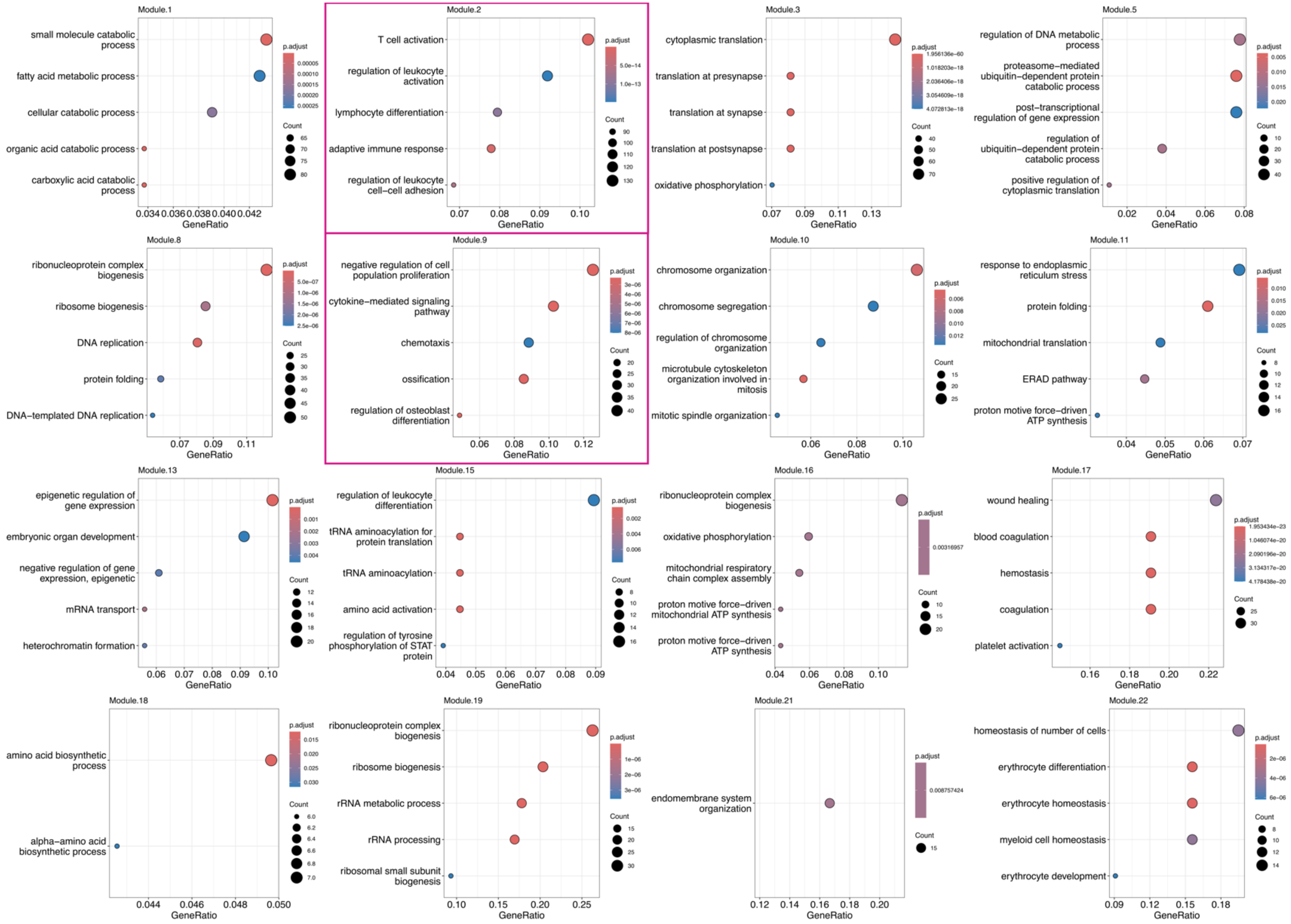

Fig S6. Gene ontology enrichment maps for each of the modules of coexpressed genes that were associated with stimulation. Modules for which statistical analyses are presented in detail in main text are indicated by frames.

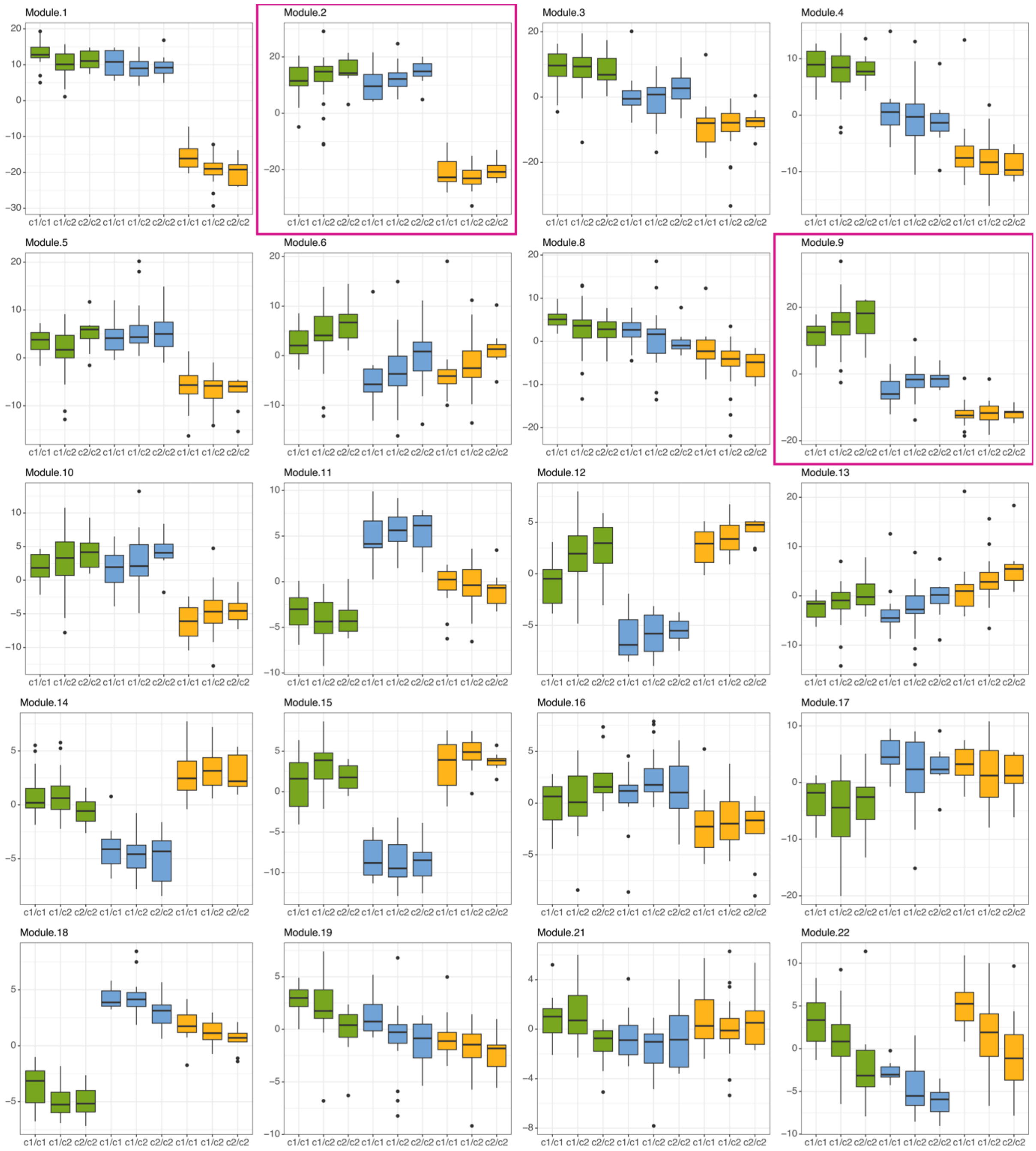

Figure S7. Effects of *Tlr2* genotype on expression of each module of coexpressed genes in splenocytes stimulated with *B. afzelii* (green), *S. pyogenes* (blue) and unstimulated controls (orange). Only modules associated with stimulation are shown. Modules for which statistical analyses are presented in detail are indicated by frames.
